# Human fibroblasts facilitate the generation of iPSCs-derived mammary-like organoids

**DOI:** 10.1186/s13287-022-03023-7

**Published:** 2022-07-28

**Authors:** Xueqin Dai, Xinye Wang, Chuanyu Yang, Maobo Huang, Zhongmei Zhou, Ying Qu, Xiaojiang Cui, Rong Liu, Ceshi Chen

**Affiliations:** 1 Key Laboratory of Animal Models and Human Disease Mechanisms of the Chinese Academy of Sciences and Yunnan Province, KIZ‐CUHK Joint Laboratory of Bioresources and Molecular Research in Common Diseases, Kunming Institute of Zoology, Chinese Academy of Sciences, Kunming, 650223 Yunnan China; 2 Kunming College of Life Science, University of Chinese Academy of Sciences, Kunming, 650223 Yunnan China; 3 Biomedical Research Center, The First Hospital of Kunming (The Affiliated Calmette Hospital of Kunming Medical University), Kunming, 650224 China; 4 Department of Surgery, Cedars-Sinai Medical Center, Samuel Oschin Comprehensive Cancer Institute, 8700 Beverly Boulevard, Davis Building 2065, Los Angeles, CA 90048 USA; 5 Translational Cancer Research Center, Peking University First Hospital, Beijing, 100034 China

**Keywords:** iPSCs, Mammary-like organoids, Fibroblasts, Breast reconstruction

## Abstract

**Background:**

Breast cancer is the most common malignancy in women worldwide, and its treatment largely depends on mastectomy. Patients after mastectomy suffer from crippled body image, self-esteem, and quality of life. Post-mastectomy breast reconstruction can improve patients’ psychosocial health. Although silicone and fat have been widely used for breast reconstruction, they have remarkable limitations. Our study aimed to establish an improved method for breast reconstruction from human-induced pluripotent stem cells (iPSCs).

**Methods:**

We used a two-step procedure to induce mammary-like organoids (MLOs) from iPSCs and applied transcriptome sequencing to analyze the gene expression profiles during the development process from embryoid bodies (mEBs) to MLOs. Moreover, we evaluated the in vitro effect of fibroblasts cell line HFF (human foreskin fibroblasts) on the size and morphology of MLOs and explored the in vivo effect of HFF on regeneration rate of MLOs.

**Results:**

MLOs had a similar gene expression profile and morphogenesis as the normal mammary glands. Furthermore, the addition of HFF increases the branching ratio and organoid diameters and facilitates the formation of multiple cell layers duct-like structures in MLOs in vitro. Finally, orthotopical transplantation of the MLOs to cleared mammary gland fad pad of NSG mice showed that HFF increases the formation of mammary gland-like structures.

**Conclusions:**

Fibroblasts facilitate iPSC-derived MLOs to generate mammary gland-like structures in both in vitro and in vivo conditions. Our findings lay a foundation for breast reconstruction by using iPSCs.

**Supplementary Information:**

The online version contains supplementary material available at 10.1186/s13287-022-03023-7.

## Background

Breast cancer is a major public health problem and life-threatening disease for females. Among all cancers in females, breast cancer ranks the first in incidence and the second in mortality [1]. Currently, clinical treatment of breast cancer includes surgery, chemotherapy, endocrine therapy, targeted therapies, and immunotherapy. Among these, surgical resection remains the primary approach [2, 3]. The breast cancer patients after mastectomy without reconstruction have worse satisfactions about body image, sexual function, and quality of life [4, 5] compared to those with reconstitution. Thus, post-mastectomy breast reconstruction (PMBR) in breast cancer patients plays a critical role in their psychosocial adjustment and physical and mental health [6]. Moreover, PMBR is also significant for females that undergo preventive mastectomy [6]. Clinically, prosthetic reconstruction using silicone and autologous reconstruction using skin, muscle, and fat are two widely used methods for PMBR. However, these methods bring high risks for several complications, including inflammation and necrosis [7–9]. Therefore, there is an urgent need to improve PMBR.

Organoids are three-dimensional in vitro culture tissues derived from stem cells or progenitors and recapitulate key structural and functional properties of in vivo organs [10]. During the past few years, organoids derived from various human tissues including the lung, liver, heart, kidney, brain, ovary and retina have been established [10, 11]. The organoid technology is helpful for mechanistic understanding of development biology, stem cell biology, cancer biology, drug safety and efficacy testing, organ replacement therapy, and personalized therapy [12–14]. In practice, patient-derived induced pluripotent stem cells are promising for generating organoids because iPSCs can be generated directly from terminally differentiated cells and can give rise to multiple cell types, thereby providing the same genetic background [15]. For example, cerebral organoids derived from iPSCs of Miller–Dieker syndrome patients have helped us to understand the cellular pathogenesis of human neurodevelopmental disorders [16]. Furthermore, cerebral organoids could recapitulate the development of embryonic brain and neocortex in vitro [17, 18]. Retinal organoids derived from iPSCs with *RPGR* gene mutation from retinitis pigmentosa patients not only recapitulated the pathogenesis but also provided proof-of-concept evidence for targeted gene therapy by correction of *RPGR* mutations [19]. In addition, kidney organoids differentiated from iPSC of individuals affected by inherited renal disease show a ciliopathic renal phenotype and illustrates dysfunctional cellular pathways underlying its pathogenesis [20].

Previously, we have shown that human iPSCs can be induced to differentiate into mammary-like organoids (MLOs) by using a reliable two-step protocol in vitro [21]. These organoids expressed common breast (luminal and basal) markers, including estrogen receptor (ER), and could be induced to produce milk protein [21]. Whether human iPSCs can be differentiated into MLOs in vivo remains unclear. In addition, it is well known that microenvironment plays important roles in the development of mammary gland [22]. The mature mammary gland contains multiple types of cells, mainly including epithelial, fibroblasts, adipose, immune, and vascular cells [23]. Among these cells, fibroblasts play important roles in mammary gland development and function via synthesizing numerous growth factors, proteases, and extracellular matrix (ECM) components that support epithelial cell survival, influence mammary gland morphogenesis and function [24, 25]. Whether fibroblasts facilitate the generation of MLOs from iPSCs remains elusive.

In this study, we investigated whether human iPSCs can be differentiated into MLOs in vivo and whether such differentiation can be improved by adding fibroblasts. Our study found that human iPSCs can be differentiated into MLOs in immunodeficient NSG mice and human fibroblasts can increase the volume and branching of MLOs both in vitro and in vivo.

## Materials and methods

### hiPSC and HFF culture, mEBs, and mammary-like organoid differentiation

Fibroblast-derived iPSCs lines were generous donated by professor Duanqin Pei’s Lab (Westlake University). The BT fibroblasts isolated from human skin tissues were reprogramed to obtain iPSCs, which were further characterized by AP staining, immunofluorescence, bisulfate sequencing, karyotyping, and teratomas formation test [26]. iPSCs were maintained using Matrigel (#35427, BD) matrix and mTeSR1 medium (#85851, Stem cell) that needed replaced fresh medium daily. HFF (human foreskin fibroblasts) (obtained from ATCC, CRL-2429) were maintained in DMEM (C11995500BT, Gibco) supplemented with 10% fetal bovine serum (10099-141, Gibco). The iPSCs were dissociated by cell dissociation solution ReLeSR (#05872, Stem cell) and suspended using complete MammoCult medium (#05261, Stem cell) with 10 μmol/L Rock inhibitor Y27632 (#Y0503, Sigma) and then seeded in 96-well plate for mEBs differentiation. The plate was put into centrifuge at 500 g at 4 °C for 7 min before put into 37 °C incubator. The mEBs were removed to 6-well ultra-low attachment plates after 24-h culture. The mEBs were collected to 3D culture that embedding mEBs in mixed Matrigel (10 mg/ml) and collagen I (4 mg/ml) (#5135, BioMatrix) with a 3:1 proportion after 10 days of suspension culture. The additional EpiCult B medium (#05602, Stem cell) adding with parathyroid hormone (pTHrP, 100 ng/ml, #1000950, PeproTech) was added into plate for 10 days. The next stage was cultured in EpiCult B medium with HGF (50 ng/ml, #10039H, PeproTech), FGF10 (50 ng/ml, #1002625, PeproTech), hydrocortisone (1 μg/ml, #H00888, Sigma), and insulin (10 μg/ml, #10365, Biogems) for 15 days. The complete MammoCult medium and EpiCult B medium were changed every 3 days.

### Immunofluorescence staining

The mEBs and organoids were fixed with 4% paraformaldehyde overnight at 4 °C, permeabilized with 0.2% Triton X-100 in 1 × PBS for 30 min, then blocked with 10% bovine serum albumin for 30 min at room temperature, and then washed three times with 1 × PBS for 5 min at the end of each step in 200-μl centrifuge tubes. The mEBs and organoids were incubated with primary antibodies at 4 °C overnight. After washing with 1 × PBS, the mEBs and organoids were incubated with secondary antibodies for 2 h at room temperature. The Hochest and DAPI were incubated to staining nuclei for 30 min at room temperature following three times wash with 1 × PBS. The images were taken using High-Resolution Fluorescence Microscopy System (Carl Zeiss, LSM880, Germany).

### Immunohistochemical staining

The immunohistochemical staining detailed procedures were carried out as described previously [27]. In brief, the fourth mammary glands were fixed in 4% paraformaldehyde, then embedded by paraffin and sectioned (5 μm) for immunohistochemical staining (IHC). The IHC staining was performed according to manufacturer’s manuals (Vector Laboratories, Burlingame, CA, USA).

### Whole mount staining

After surgically removing the 4th and 5th pairs of mammary glands from the anaesthetized NSG mice, mammary-like organoids were orthotopically transplanted to the mammary gland-cleared fat pad. Two months later, the mammary glands were collected and spread on glass slide and fixed in Carnoy's fixative for 2 to 4 h at room temperature. After washing in 70% ethyl alcohol for 15 min, tissues were stepwise hydrated by 60%–40%–20% ethyl alcohol and distilled H_2_O, 15 min for each step. Tissues were stained in carmine alum over night at room temperature and then washed in 70%–90%–100% ethyl alcohol to dehydrate the tissue, 15 min each step. Tissues were cleared in xylene for 48 h and mounted with Permount. Images were taken for documentation and further analysis.

### Western blotting and RT-qPCR analysis

RIPA lysis buffer supplemented with 0.2% protease inhibitor P8340 was used to lyse iPSCs and mammary-like organoids for 30 min on ice. After protein extraction, around 20 μg proteins per sample was subjected to 10% SDS-PAGE and blotted to PVDF membranes (IPVH00010, Millipore). The membranes were incubated with primary antibodies overnight at 4 °C, followed by incubating with secondary antibodies for 1 h at room temperature. Finally, the membranes were incubated with Western chemiluminescent HRP substrate (Millipore Sigma, Burlington, MA, USA), and proteins were detected on an LAS-4000 Imaging system (GE, PA, USA). The antibodies information of immunofluorescence staining, immunohistochemical staining, and Western blotting is listed in Additional file 1: Table S1.

Total mRNA of iPSCs and mammary-like organoids were isolated using TRIzol® reagent (15590-026, Invitrogen). Reverse transcription was performed using the HiScript III RT SuperMix for qPCR (+ genomic DNA wiper) kit (R323, Vanzyme), and gene expression was quantified using SYBR Green (4472908, Applied Biosystems) on the ABI-7300 system (Applied Biosystems). The primer sequences for RT-qPCR are listed in Additional file 1: Table S2.

## Results

### In vitro differentiation of human iPSCs into MLOs

**Fig. 1:**
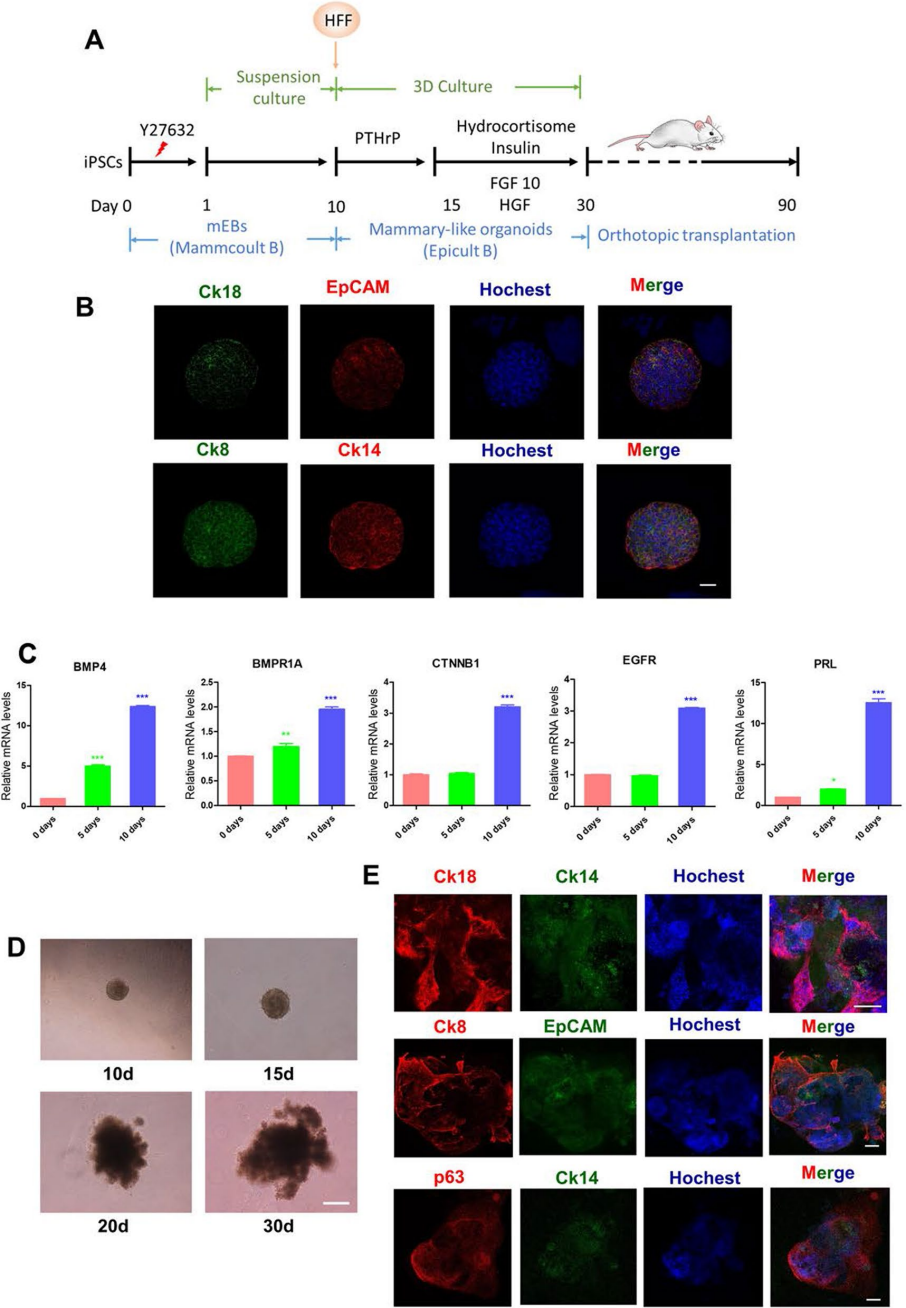
Establishing MLOs from human iPSCs. **A** Diagram depicting the generation of MLOs from human iPSCs using optimized media. **B** Immunofluorescence analysis of the expression of luminal and basal epithelial markers in 10-day mEBs. Bar, 50 μm. **C** RT-qPCR analysis of the expression of mammary development-related genes in mEBs from day 0 to day 10. Statistical significance was determined using one-way ANOVA with a Tukey posttest correction. RT-qPCR have been repeated at least three times. Data are depicted as mean ± SD. *p < 0.05, **p < 0.01, ***p < 0.001. **D** Bright-field images depicting major MLOs phenotypes from day 10 to day 30 (d: days). Bar, 50 μm. **E** Immunofluorescence analysis of epithelial markers in 30-day MLOs. Bar, 200 μm. All experiments have been repeated for at least twice

We engaged a two-step procedure [21] to induce MLOs from human iPSCs in vitro. The in vitro induced differentiation was performed according to the processes shown in Fig. 1A. The stemness and pluripotency status of human iPSCs were confirmed by detecting the expression of SOX2, OCT4, and SOX9 using Western blotting (Additional file 1: Figure S1A). To obtain medium-cultured embryoid bodies (mEBs), human iPSCs were cultured in suspension with MammoCult medium for 14 days (Additional file 1: Figure S1B). We detected the expression of non-neuron ectoderm markers (p63, CK8, and CK18), meso-marker (T/Brachyury), and pluripotent marker (SOX2) in mEBs at days 5, 7, 10, and 14 of culture, respectively. The results showed that, after 10-day culture, the expression of p63, CK8, and CK18 was maintained, T/Brachyury tended to decrease, and SOX2 decreased sharply in mEBs (Additional file 1: Figure S1C). These data suggest that day 10 appears to be an optimal time point to induce mEBs to differentiate into organoids. To confirm this, we performed immunofluorescence (IF) assays to examine the expression of epithelial markers (CK18, EpCAM, CK8, and CK14) in 10-day mEBs and found they were highly expressed (Fig. 1B). In addition, the transcriptional expression of several genes that are critical for mammary development pathways (BMP4, BMPR1A, CTNNB1, EGFR, and PRL) was induced stepwise from day 0 to day 5 and peaked at day 10 (Fig. 1C).

Next, we cultured the 10-day mEBs in 3D Matrigel/Collagen I mixed gel and EpiCult-B medium for another 20 days. To induce mEBs to differentiate into MLOs, PTHrP was added from days 10 to 15, hydrocortisone, insulin, FGF10 and HGF were added from days 15 to 30 (Fig. 1A). As shown in Fig. 1D, the branch and alveoli-like structure in organoids were emerged after 15 days. After being cultured for 30 days in total, MLOs were collected for IF assay. The results showed that these MLOs expressed mammary markers (CK18, CK14, CK8, EPCAM, and p63) and generated alveoli-like structures (Fig. 1E). Taken together, our results demonstrated that MLOs could be induced efficiently from iPSCs in vitro.

### Transcriptome analysis during MLOs differentiation

**Fig. 2:**
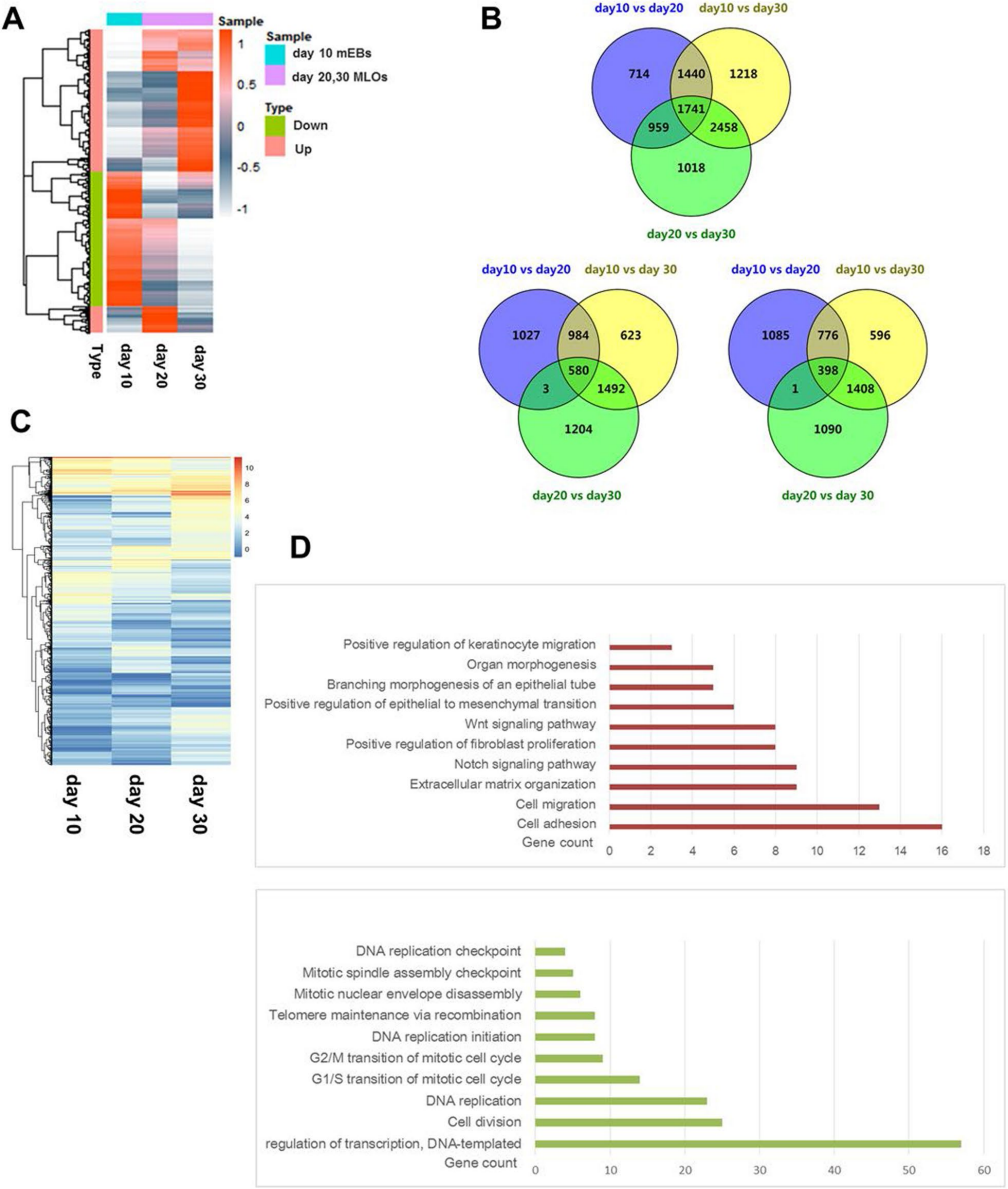
RNA sequencing of mEBs and MLOs. **A** Cluster heat map of differentially expression profiles of mRNAs of cultured MOLs collected at indicated time points. RNA sequencing data from 10-day mEBs, 20- and 30-day MLOs were analyzed. The heat map included 5627 differentially expressed gene. Corrected *P* value of 0.05 and absolute fold change of 1.5 were set as the threshold for significantly differential expression. **B** Venn diagram showing differentially expressed genes overlapped between different days of culture during MLOs development. There were 1741 genes expression changed during the differentiation (up panel). Among these genes, 580 genes (left panel) were upregulated and 398 genes (right panel) were downregulated. Corrected *P* value of 0.05 and absolute fold change of 1.5 were set as the threshold for significantly differential expression. **C** The overlap gene heat map of differentially expressed genes in figure (**B**). **D** The GO-BP analysis of the overlapped genes-related biological process during MLOs development

To provide a comprehensive and unbiased assessment of whether these mEBs did differentiate toward MLOs, and to identify genes/signaling that contribute to mammary gland development, we performed RNA sequencing (RNA-seq) analysis in 10-day mEBs and 20-/30-day MLOs. We observed a global reprogramming of gene expression during the differentiation from mEBs to MLOs (Fig. 2A). After overlapping the RNA sequencing data from 10-day mEBs, 20- and 30-day MLOs, we found that the expression of 1741 genes changed (Fig. 2B). Among these genes, 580 genes were upregulated and 398 genes were downregulated (Fig. 2B-C). Gene ontology biological process (GO-BP) analysis revealed that ten pathways related to mammary development were upregulated, such as organ morphogenesis, branch morphogenesis of an epithelial tube, and Wnt and BPM signaling (Fig. 2D). Meanwhile, most of the downregulated genes were enriched in processes related to terminal differentiation, such as cell division and cell cycle regulation, telomere maintenance, and transcription regulation (Fig. 2D).

**Fig. 3:**
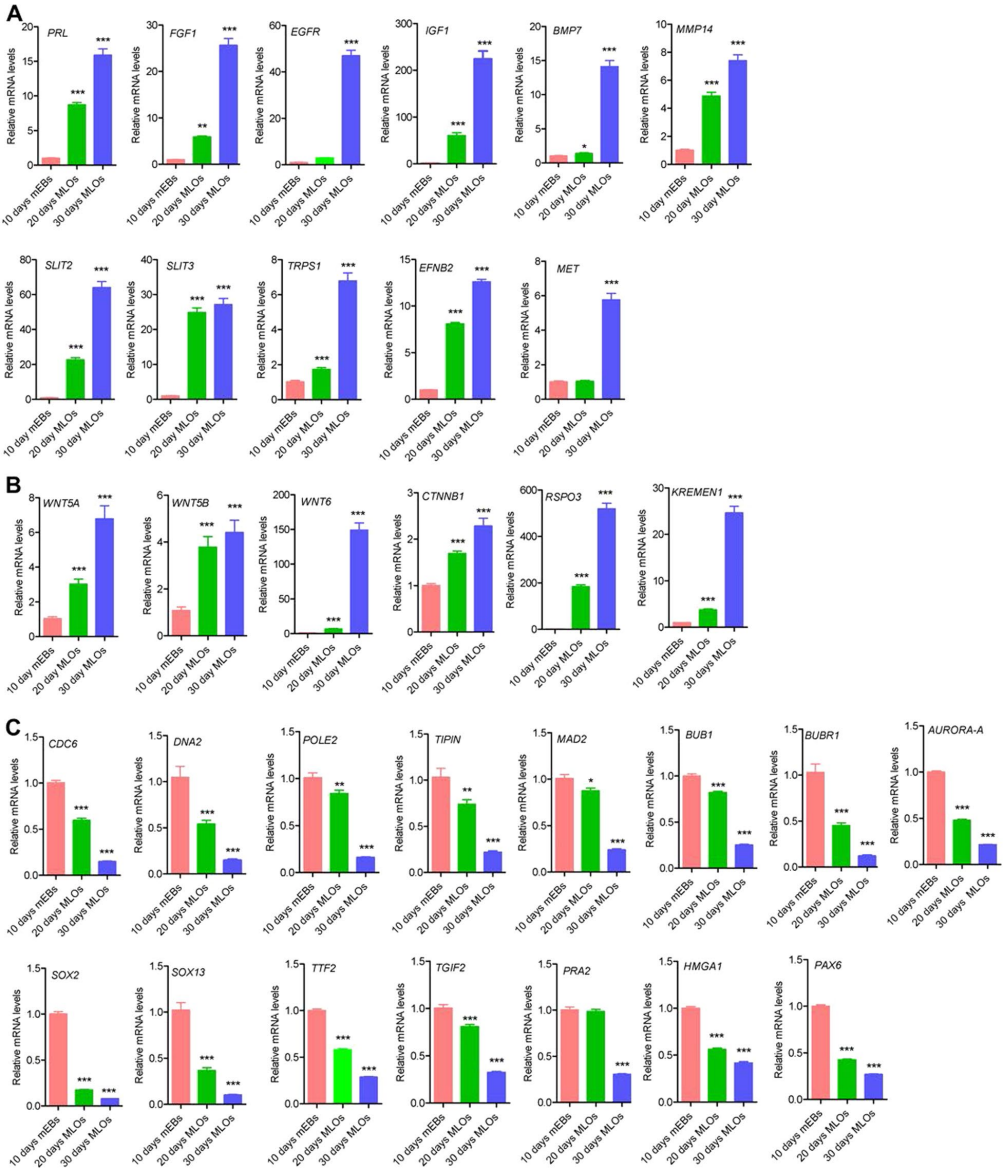
Mammary gland development-associated genes’ expression during MLOs formation. **A** The expression of genes in mammary gland morphogenesis and branching morphogenesis of epithelial tube increased gradually during MLOs generation; **B** The expression of genes involved in Wnt signaling pathway increased gradually during MLOs formation; **C** the expression of genes involved in cell division, cell cycle, regulation of transcription and telomere maintenance decreased gradually during MLOs generation. Statistical significance was determined using one-way ANOVA with a Tukey posttest correction. RT-qPCR have been repeated at least three times. Data are depicted as mean ± SD. *p < 0.05, **p < 0.01, ***p < 0.001

Next, we confirmed the expression profiles of several key genes in these processes by reverse transcription-quantitative polymerase chain reaction (RT-qPCR) (Fig. 3). The results showed that 11 genes (*PRL, FGF1, EGFR, IGF1, BMP7, MMP14, SLIT2, SLIT3, TRPS1, EFNB2*, and *MET*) involved in the regulation of mammary gland morphogenesis (Fig. 3A) and 6 genes (*WNT5A, WNT5B, WNT6, CTNNB1, RPO3,* and *KREMEN1*) of Wnt signaling (Fig. 3B) increased gradually during the differentiation process from mEBs to MLOs. In contrast, 15 genes (*CDC6, DNA2, POLE2, TIPIN, MAD2, BUB1, BUBR1, AURORA-A, SOX2, SOX13, TIF2, TGIF2, PRA2, HMGA1,* and *PAX6*) involved in process-related cell division and cell cycle regulation, telomere maintenance, and transcription regulation were declined step-wisely during MLO development (Fig. 3C). Taken together, these results not only confirmed that the iPSCs-derived organoids were MLOs, but also revealed that genes involved in several signaling pathways, such as Wnt and Notch signaling, might play important roles in human mammary gland development.

### The generation of MLOs is improved by human fibroblasts

**Fig. 4:**
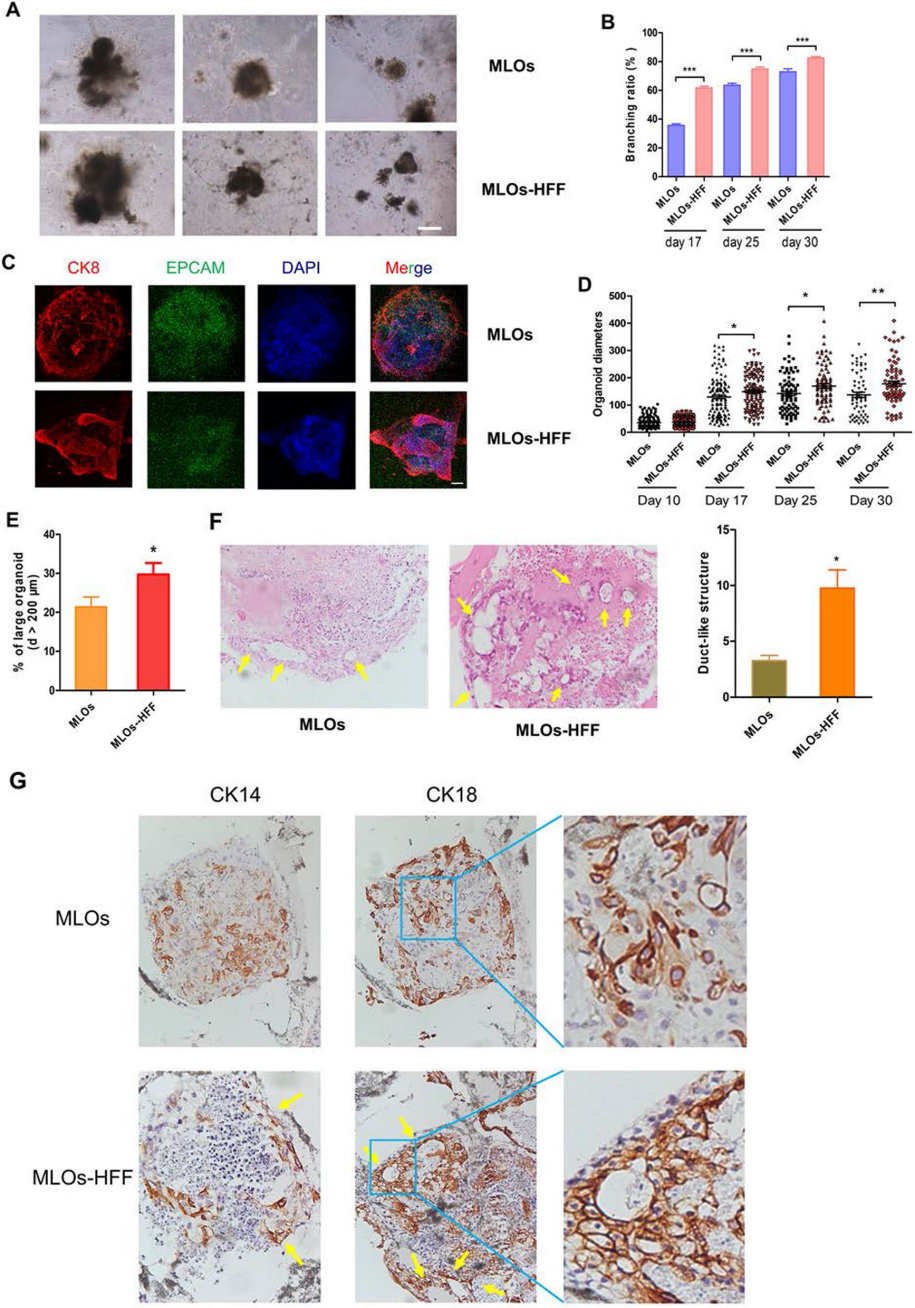
Establishing MLOs from human iPSCs is improved by HFF. **A** Representative bright-field images of MLOs generated from human iPSCs with (lower panel) or without HFF (upper panel) at day 30. Bar, 50 μm. **B** Branching ratios of MLOs and MLOs-HFF. **C** Representative immunofluorescence images of MLOs and MLOs-HFF at day 30. Bar, 100 μm. **D** Sizes of MLOs and MLOs-HFF at different days. Day 10: MLOs (*n* = 136), MLOs-HFF (*n* = 107); day 17: MLOs (*n* = 131), MLOs-HFF (*n* = 121); day 25: MLOs (*n* = 83), MLOs-HFF (*n* = 84); day 30: MLOs (*n* = 66), MLOs-HFF (*n* = 78). **E** Percentage of large organoids (diameter > 200 μm) of MLOs and MLOs-HFF at day 30. **F** Representative hematoxylin and eosin staining of MLOs and MLOs-HFF at day 30 (left); percentage of organoids displaying duct-like structure in MLOs (*n* = 13) and MLOs-HFF (*n* = 39) at day 30 (right). **G** Representative immunohistochemical staining of MLOs and MLOs-HFF at day 30. Two-tailed Student t test, *p < 0.05, **p < 0.01, ***p < 0.001

Fibroblasts are critical in normal mammary gland development [25]. To explore whether human fibroblasts would facilitate the generation of MLOs, HFF were added into 10-day mEBs (Fig. 1A and Additional file 1: Figure S2A). The result showed that the branching ratios of MLOs at days 17, 25, and 30 were increased with the addition of HFF (referred as MLOs-HFF), when compared with MLOs without HFF (referred as MLOs) (Fig. 4A-B). In addition, the addition of HFF conditional medium to MLOs was unable to promote branch formation (Additional file 1: Figure S2B). We performed IF assays and found that MLOs-HFF generated more alveoli-like structures than MLOs and the alveoli-like structures in MLOs-HFF (Fig. 4C) are morphologically more similar to organoids formed from mammary epithelial cells in vitro [28]. Meanwhile, we found that MLOs-HFF had a significantly larger volume than MLOs at days 17, 25, and 30 (Fig. 4D). In addition, MLOs-HFF had a higher proportion of large organoids (diameters > 200 μm) than MLOs (Fig. 4E).

We analyzed the structure of MLOs by H&E staining and found that MLOs-HFF generated more duct-like structures than MLOs (Fig. 4F). Furthermore, the duct-like structures in MLOs-HFF contain multiple cell layers, which is morphologically more similar to human mammary ducts, whereas duct-like structures in MLOs typically had single cell layer (Fig. 4G). Taken together, our results implicated that HFF could facilitate the generation of MLOs from iPSCs.

### Functional validation of MLOs and MLOs-HFF in NSG mice

**Fig. 5:**
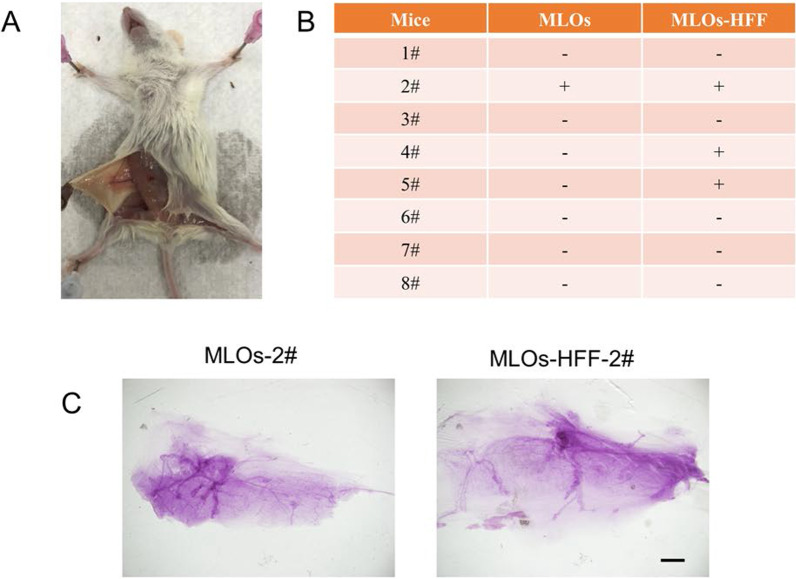
Functional validation of MLOs and MLOs-HFF in NSG mice. **A** Representative image of orthotopical transplantation of MLOs and MLOs-HFF into the fad pad of NSG mouse. **B** Results of orthotopical transplantation of MLOs and MLOs-HFF. “+” means mammary glands have regenerated; “−” means no mammary glands were regenerated. **C** Representative whole-mount staining of regenerated mammary glands. Scale bar: 20 μm

To investigate whether HFFs also facilitate mammary development in vivo, we conducted the orthotopical transplantation of 30-day MLOs and MLOs-HFF to mammary gland-cleared fat pad of NOD-SCID IL2rg null (NSG) mice (Fig. 1A), which are severe combined immunodeficient mice suitable for transplantation and developmental studies [29, 30]. We transplanted 10 mature MLOs or MLOs-HFF to each fad pad (*n* = 8) and detected the mammary regeneration rate 2 months after transplantation (Fig. 5A). The results showed that while 37.5% (3/8) of NSG mice received MLOs-HFF regenerated mammary-like structures, only 12.5% (1/8) of NSG mice received MLOs developed mammary-like structures (Fig. 5B). The regenerated mammary-like structures were confirmed by whole mount staining based on histological analysis (Fig. 5C). Together, our results demonstrated that the human iPSCs-derived MLOs have potential to generate mammary-like structures and HFF can facilitate such potentials.

## Discussion

Previously, we have developed a two-step protocol to generate MLOs from human iPSCs, and such MLOs express breast-specific, luminal and basal markers and mainly contain epithelial cells [21]. However, whether MLOs can be regenerated into mammary glands and, if so, whether this process can be improved by other mesenchymal cells, such as fibroblasts, remained unclear. The normal mammary gland development needs epithelial cells’ communication with the cellular microenvironment and extracellular matrix [31]. In our present system, we found MLOs can be regenerated into mammary gland-like structure and HFF can facilitate MLOs’ generation and branch formation.

Currently, the most widely used methods for PMBR include prosthetic reconstruction using silicone and autologous reconstruction using skin, muscle, and fat. However, limitations of these methods largely reduced patient satisfaction. For example, the implantation of silicone is limited by infection, implants migration and rupture, and the need for continually replacement [32]. The autologous includes fat and tissue grafting. Autologous fat grafting is restricted to small volume, unpredictable reabsorption of the graft and adverse events after grafting [33]. Autologous tissue grafting also has disadvantages in the morbidity of surgical site, such as inflammation, tissue necrosis, and septicemia [34, 35]. The adipose tissue engineering in combination with hydrogels provides a new insight in post-mastectomy breast regeneration [36], but it may be limited by the small volume of grafting adipose tissue. A preferred method for PMBR should have the characteristics of fewer traumas, less postoperative side effects, high biocompatibility, good sustainability, and high appearance restoration. The breast reconstruction methods used currently are unable to satisfy all of these characteristics. The breast epithelial cells in MLOs induced by human iPSCs may improve the biocompatibility, decrease the donor site and transplant site morbidity, and avoid secondary absorption like fat grafting.

The development of normal human mammary glands is controlled by cytokines, steroids, and peptide hormones during adolescence and menstrual cycle. BMP signaling and Wnt/β-catenin signaling play critical roles in mammary gland development [37]. In line with this, BMP4 and CTNNB1 expression were dramatically increased in mEBs development (Fig. 1C). GO-BP analysis (Fig. 2D) and RT-qPCR (Fig. 3A-B) results illustrated that BMP7, Wnt 5A, Wnt 5B, Wnt6, CTNNB1, and several other factors in BMP signaling and Wnt/β-catenin pathways were increased during MLOs differentiation. The Notch signaling is important in mammary epithelial cell differentiation during development and mammary gland stem cell niche [38]. In consistent with this, the Notch signaling, such as JAG2 and MAML2, was enriched in the process of MLOs formation. Several other cellular pathways, for example, the positive regulation of keratinocyte migration, organ and branching morphogenesis and the positive regulation of epithelial to mesenchymal transition and cell migration, were also enriched during MLOs formation (Fig. 2D). Taken together, these genes may play vital roles in ensuring the differentiation and architecture of MLOs.

There are multiple types of cells in the microenvironment of mammary glands, including fibroblast, macrophage, adipocytes, endothelial cells, and other immune cells [39]. To mimic the normal mammary cellular components, three types of mesenchymal cells (HFF, mesenchymal stem cell line qMSC, vascular endothelial cell line HUVEC) were added individually or in combination during the 3D culture process of the MLOs induced from mEBs. We found that neither MSC nor HUVEC addition had obvious effects on MLO generation (Additional file 1: Figure S3), while HFF addition significantly promoted MLOs formation. These results demonstrate that our MLOs system can be used to study the roles of cellular components in breast development. In combination of single-cell sequencing and gene editing techniques, our system may be used to investigate the molecular processes underlying breast carcinogenesis from chemical and physical environment exposure.

Currently, the mechanisms by which HFF promote MLOs remain elusive. We tried to understand why addition of HFF can facilitate the mammary-like organoids structural integrity in vitro. We found that the mammary epithelial markers CK14 and EPCAM expression were increased in MLOs-HFF at day 30 (Additional file 1: Figure S4). These results suggest that HFF may facilitate the formation of multiple-cell-layer duct-like structures, the extension and branching of MLOs by promoting the proliferation of mammary epithelial cells. But more evidence is needed to validate this speculation. The HFF conditional medium was concentrated about 150 times and was added to organoids during 3D culture. HFF conditional medium had no significant impacts on organoid formation and branching after 30 days of culture (Additional file 1: Figure S2B). We also tried to culture the organoids together with HFFs which were cultured in 0.4-μm bore diameter transwells, which also did not show obvious facilitation (data not shown). Thus, these results indicated that factors released from HFF may not be the major players in HFF-induced MLOs development. Instead, direct cell–cell attachment and interaction between HFFs and MLOs may be responsible for promoting MLOs development and branch formation. It is well known that several signaling pathways, such as Notch and Eph, require cell–cell interaction [40, 41]. It would be interesting to further discover the underlying mechanisms by which HFFs promote MLOs formation in the future. The HFF used in our study is an immortalized human fibroblast cell line, which is easy to obtain and culture in vitro. In future, we plan to compare the influences of different kinds of fibroblast cell lines and ex vivo fibroblasts from skin biopsy sample on MLOs generation.

Although iPSCs-derived organoids possess enormous potent in regenerative and precision medicine, a number of hurdles should be overcome in order to enable a more effective disease and development modeling. For example, immature embryonic or fetal identity is found to be persistent in iPSCs-derived organoids [42]. One approach to improve the maturity level of organoids in vitro is increasing the cultivation times. Besides, engraftment of organoids to immunocompromised murine hosts that formed “humanized” chimeric may enhance maturation due to integration of microenvironment factors [43]. Another hurdle is that current organoids differentiation heavily relies on self-organization, which will cause heterogeneity at cellular scale and variation at organoids shapes and sizes [44]. Synergistic techniques of iPSCs-derived organoids and organ-on-chip may partially overcome variability in organoids shapes and sizes [44]. Finally, unlike in vivo organs, iPSCs-derived organoids lack the supporting tissues (e.g., vasculature, immune and nervous system) and microenvironment [44, 45]. In order to mimic the normal development microenvironment of human organs, some special cell types should also be added during the generation of organoids. In the present study, we indeed demonstrated that HFF could facilitate MLO development in both in vitro and in vivo system.

## Conclusion

In summary, we determined the gene expression profile during the differentiation process of mEBs to MLOs. We found the effect of HFF in increasing the branching ratio, organoids diameters, and multiple cell layers’ duct-like structures formation of MLOs in vitro and HFF could increase the mammary gland-like regeneration rate in orthotopical transplantation in NSG mice. The regeneration rate is still not high enough, and other types of cells, such as immune cells, might also be required to improve the regeneration efficiency in vitro and in vivo. This study provides a potential implication to PMBR of breast cancer patients and plastic surgery patients.

## Supplementary Information

**Additional file 1. Figure S1**. Differentiation of human iPSCs into mEBs. (Related to Figure 1) (A). The morphology (left panel) and expression of pluripotency markers (right panel) of human iPSCs. Bar, 50 μm. (B). The morphology of mEBs after 3, 5, 7 and 10 days of suspension culture (d: days). (C). The expression profiles of different markers in mEBs after 5, 7, 10 and 14 days’ culture.** Figure S2**. HFF facilitates MLOs branching. (Related to Figures 1 and 4) (A). Representative bright-field images of MLOs-HFF (left panel). HFF expressing green fluorescent protein (GFP) was shown in MLOs (middle panel). HFF expression fibroblast marker α-SMA (α-Smooth Muscle Actin) and vimentin were detected (right panel). Bar, 50 μm. (B). Representative bright-field images of MLO generated from human iPSCs (left), human iPSCs with HFF (center) and human iPSCs with HFF conditioned medium (right). Bar, 50 μm.** Figure S3**. Co-culture of MLOs with mesenchymal cells. (A). Co-culture of mEBs with different mesenchymal cells. Bar, 500nm. (B). The organoids displayed increased branching structures after adding HFF alone or all of three mesenchymal cell lines. Bar, 100 μm.** Figure S4**. HFF promotes MLOs differentiation. The differential expression profiles of mammary epithelial markers between MLOs and MLOs-HFF after 30 days’ culture (d: days). The expression of CK 14 and EPCAM were increased in MLOs-HFF.** Table S1**. Antibody list related to Figure 1, S1, 4and S4.** Table S2**. The list of primer in RT-qPCR.** Table S3**. The information ofmRNA sequencing data quality control and genome alignment.

## Data Availability

RNA sequencing row data have been uploaded and deposited to the National Center for Biotechnology Information under the BioProject accession PRJNA656090. Additional experimental details and procedures are provided in the additional files and Additional file 1: Table S3.

## Abbreviations

HFF: Human foreskin fibroblasts
MLOs: Mammary-like organoids
MLOs-HFF: Mammary-like organoids with the addition of HFF
iPSCs: Induced pluripotent stem cells
PMBR: Post-mastectomy breast reconstruction
mEBs: Embryoid bodies
IF: Immunofluorescence
RNA-seq: RNA sequencing
GO-BP: Gene ontology biological process
RT-qPCR: Reverse transcription-quantitative polymerase chain reaction
NSG mice: NOD-SCID IL2rg null mice
