# Supplementary material for: Human fibroblasts facilitate the generation of iPSCs-derived mammary-like organoids
Source: Stem Cell Res Ther. 2022 Jul 28;13:377. doi: 10.1186/s13287-022-03023-7 (PMC9330643; doi:10.1186/s13287-022-03023-7)
Supplement: Supplementary file 1 — Additional file 1. Figure S1. Differentiation of human iPSCs into mEBs. (Related to Figure 1) (A). The morphology (left panel) and expression of pluripotency markers (right panel) of human iPSCs. Bar, 50 μm. (B). The morphology of mEBs after 3, 5, 7 and 10 days of suspension culture (d: days). (C). The expression profiles of different markers in mEBs after 5, 7, 10 and 14 days’ culture. Figure S2. HFF facilitates MLOs branching. (Related to Figures 1 and 4) (A). Representative bright-field images of MLOs-HFF (left panel). HFF expressing green fluorescent protein (GFP) was shown in MLOs (middle panel). HFF expression fibroblast marker α-SMA (α-Smooth Muscle Actin) and vimentin were detected (right panel). Bar, 50 μm. (B). Representative bright-field images of MLO generated from human iPSCs (left), human iPSCs with HFF (center) and human iPSCs with HFF conditioned medium (right). Bar, 50 μm. Figure S3. Co-culture of MLOs with mesenchymal cells. (A). Co-culture of mEBs with different mesenchymal cells. Bar, 500nm. (B). The organoids displayed increased branching structures after adding HFF alone or all of three mesenchymal cell lines. Bar, 100 μm. Figure S4. HFF promotes MLOs differentiation. The differential expression profiles of mammary epithelial markers between MLOs and MLOs-HFF after 30 days’ culture (d: days). The expression of CK 14 and EPCAM were increased in MLOs-HFF. Table S1. Antibody list related to Figure 1, S1, 4and S4. Table S2. The list of primer in RT-qPCR. Table S3. The information ofmRNA sequencing data quality control and genome alignment. [file 13287_2022_3023_MOESM1_ESM.pdf]

Supplemental Information

Human fibroblasts facilitate the generation of iPSCs-derived mammary-like organoids

Xueqin Dai[1,2], Xinye Wang[1,2], Chuanyu Yang[1], Maobo Huang[3], Zhongmei zhou[1], Ying Qu[4], Xiaojiang Cui[4], Rong Liu[1,5]*, Ceshi Chen[1]*

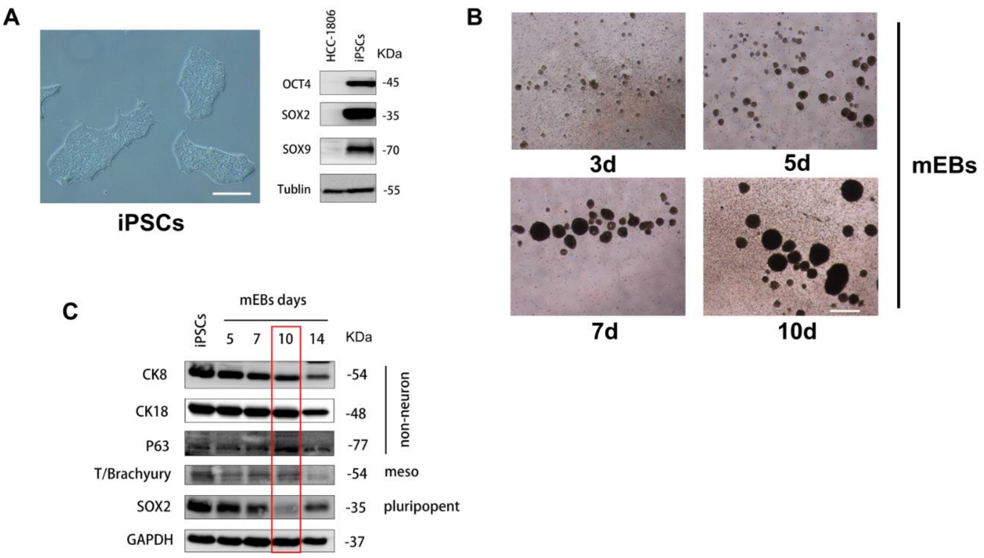

Figure S1. Differentiation of human iPSCs into mEBs. (Related to Figure 1)

(A). The morphology (left panel) and expression of pluripotency markers (right panel) of human iPSCs. Bar, 50 μm.

(B). The morphology of mEBs after 3, 5, 7 and 10 days of suspension culture (d: days).

(C). The expression profiles of different markers in mEBs after 5, 7, 10 and 14 days' culture.

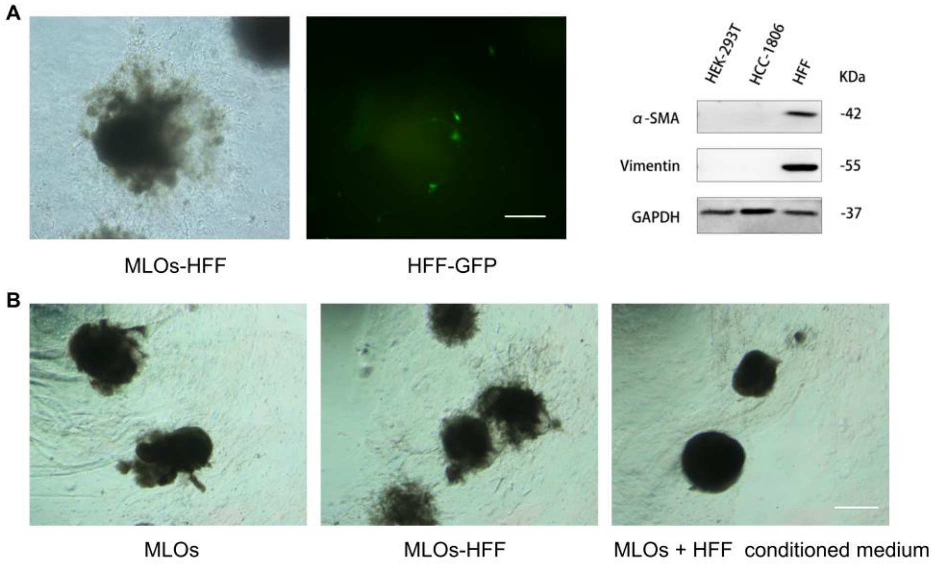

Figure S2. HFF facilitates MLOs branching. (Related to Figures 1 and 4)

(A). Representative bright-field images of MLOs-HFF (left panel). HFF expressing green fluorescent protein (GFP) was shown in MLOs (middle panel). HFF expression fibroblast marker α-SMA (α-Smooth Muscle Actin) and vimentin were detected (right panel). Bar, 50 μm

(B). Representative bright-field images of MLO generated from human iPSCs (left), human iPSCs with HFF (center) and human iPSCs with HFF conditioned medium (right). Bar, 50 μm

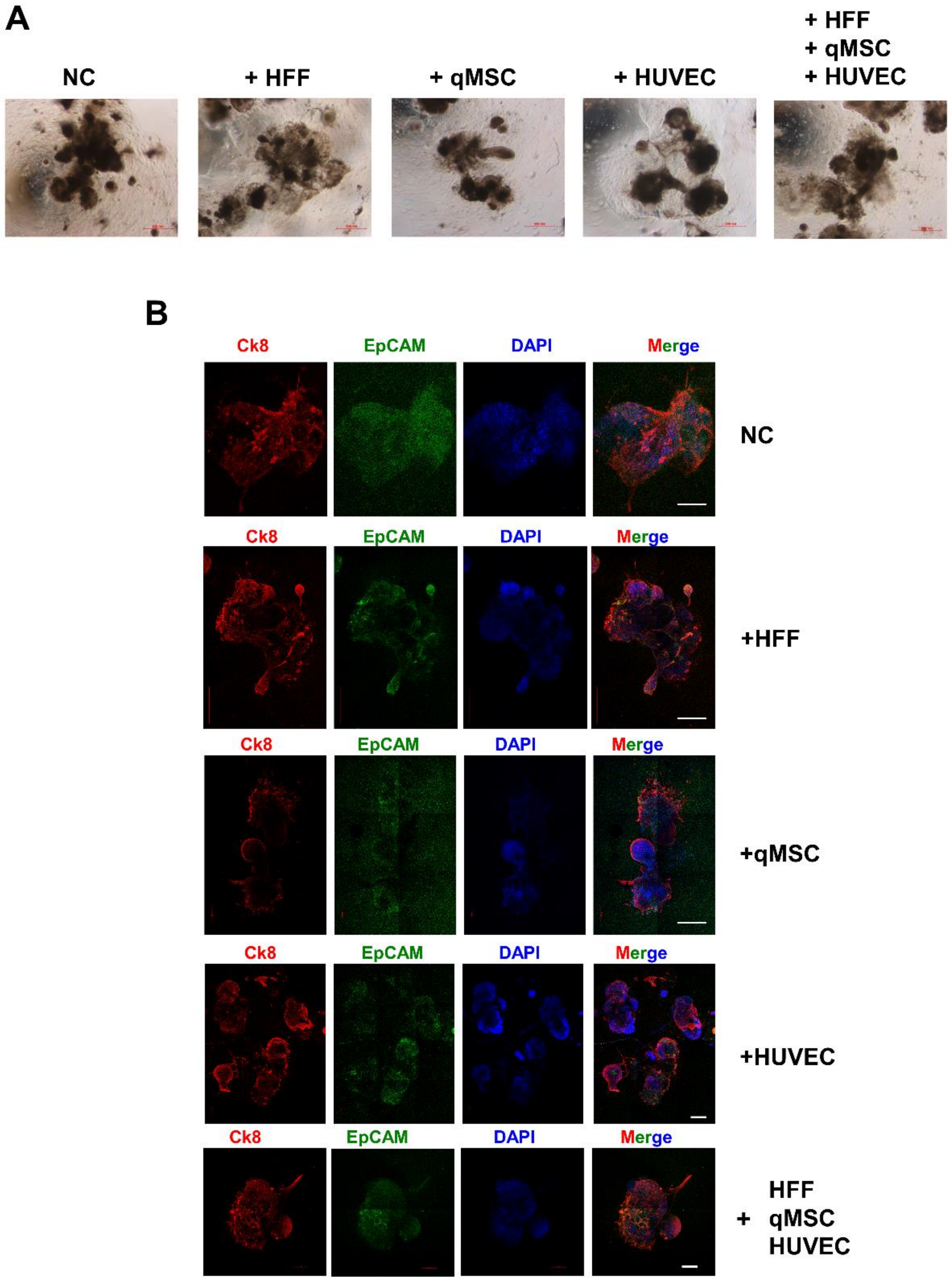

Fig S3. Co-culture of MLOs with mesenchymal cells.

(A). Co-culture of mEBs with different mesenchymal cells. Bar, 500nm.

(B). The organoids displayed increased branching structures after adding HFF alone or all of three mesenchymal cell lines. Bar, 100 μm

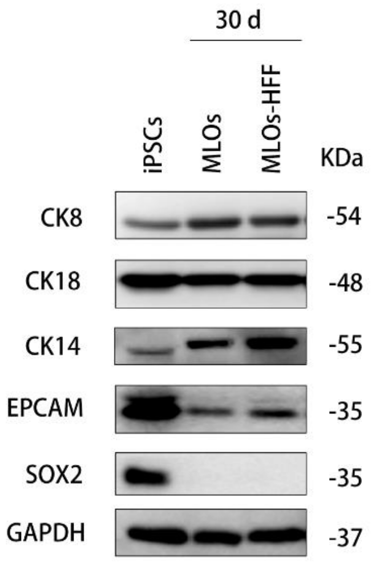

Figure S4 HFF promotes MLOs differentiation.

The differential expression profiles of mammary epithelial markers between MLOs and MLOs-HFF after 30 days' culture (d: days). The expression of CK 14 and EPCAM were increased in MLOs-HFF.

Supplemental Experimental Procedures

Image analysis

The branching ratio in Figure 4B was counting branched and total organoids number to calculation the branched organoids proportion under the inverted microscope (Nikon ECLIPSE Ti) during the differentiation process. The MLOs and MLOs-HFF in plate pictures were taking by stereoscope (SZX16, Olympus) to measuring the all organoids diameter and then to statistical ananlysis diameter change at day 10, 17, 25 and 30 (Figure 4D). The IHC duct-like structure results in Figure 4F and 4G were counting the duct in different vision zoom in 200╳ at microscope (OLYPUS CX41) then calculate the mean duct number.

mRNA sequencing and pathway analysis

MLOs total mRNA was extracted using Trizol reagent (Invitrogen, CA, USA) following the manufacturer's procedure. The total RNA quantity and purity were analysis of Bioanalyzer 2100 and RNA 6000 Nano Lab Chip Kit (Agilent, CA, USA) with RIN number >7.0. A total amount of 3 μg RNA per sample was used as input material for RNA-Seq library preparations. Sequencing libraries were generated using NEB Next Ultra II RNA Library Prep Kit for Illumina (NEB, E7760) following manufacturer's recommendations and index codes were added to attribute sequences to each sample. Samples were run on an Illumina Hi-seq instrument. Raw data (raw reads) of fastq format were firstly processed by Fastp software (0.19.3). In this step, clean data (clean reads) were obtained by removing reads containing adapter, reads containing ploy-N and low-quality reads from raw data. At the same time, Q30 and GC content of the clean data were calculated. The quality control and clean reads results were shown in the Table S3. All the downstream analyses were based on the clean data with high quality. Reference genome and gene model annotation files were downloaded from the genome website directly. Paired-end clean reads were aligned to the reference genome of Homo sapiens (RefGene, v20181115) using STAR V20201. Cufflinks v2.2.1 was used to count the reads numbers mapped to each gene. And then FPKM of each gene was calculated based on the length of the gene and reads count mapped to this gene. Differential expression genes analysis of Day 10, Day 20 and Day 30 was performed using the Cufflinks v2.2.1. The *P*-values were adjusted using the Benjamini & Hochberg method. Corrected *P*-value of 0.05 and absolute fold change of 1.5 were set as the threshold for significantly differential expression. Gene Ontology (GO) enrichment analysis of differentially

expressed genes was implemented by the clusterProfiler R package (v3.10.0), in which gene length bias was corrected. GO terms with corrected P-value less than 0.05 were considered significantly enriched by differential expressed genes.

Statistical analysis

Statistical significance for RT-qPCR data (Figures 1C and 3) was determined using one-way ANOVA with a Tukey post-test correction. The two-tailed Student t test was used to compare the gene expression difference between MLOs and MLOs-HFF (Figure 4B, D, E and F). Only differences having a P-value (two-tailed) lower than 0.05 were considered as being significant. Data are presented as the mean ± SD (standard deviation). All the Western blots (Figures S1A and C) and RT-qPCR (Figures 1C and 3) have been repeated at least three times. All statistical analyses were performed using GraphPad PRISM version 5 (GraphPad, San Diego, CA, USA).

Table S1. Antibody list related to Figure 1, S1, 4 and S4

Antigen	Type	IF	WB	IHC	Manufacturer	Cat. Num.
Ck18	Rabbit	1:250	1:2000	1:800	Abcam	ab181597
Ck8	Rabbit	1:100	1:1000	1:250	Abcam	Ab53280
Ck14	Mouse	1:400	1:100	1:50	Abcam	Ab7800
EpCAM	Mouse	1:40	1:200	1:40	Abcam	Ab8666
P63	Rabbit	1:100	1:500	-	GENETEX	GTX102425
T/Brachyury	Rabbit	-	1:1000	-	Novus	NBP2-24676
OCT4	Mouse	-	1:1000	-	Stem cell	60059
SOX2	Rabbit	-	1:1000	-	CST	3579
SOX9	Rabbit	-	1:500	-	Millipore	AB5535
GAPDH	Mouse	-	1:1000	-	Santa Cruz	Sc-32233
Tublin	Mouse	-	1:4000	-	Sigma	T5168
α-SMA	Rabbit		1:1000		CST	19245
Vimentin	Rabbit		1:1000		CST	5741

Table S2. The list of primer in RT-qPCR.

Gene	Primer
BMP4	(F) CCTGAGCCTTTCCAGCAAGT (R)ACGACCATCAGCATTCGGTT
BMPR1A	(F) AGCCAGCTGGTTTCGAGAAA (R) AGAGCTGAGTCCAGGAACCT
CTNNB1	(F)TCTTCGTCATCTGACCAGCC (R) GGGATGGTGGGTGTAAGAGC
EGFR	(F) GCTGCCTCCTGGACTATGTC (R) TGATCTTGACATGCTGCGGT
PRL	(F) ACACTTCTTCCCTTGCCACC (R) GGCTCATTCCAGGATCGCAA
FGF1	(F)CTCTACTGTAGCAACGGGGG (R) ATATACACCTCCCCCACGCT
IGF1	(F) GCTGGTGGATGCTCTTCAGT (R) CACTCATCCACGATGCCTGT
BMP7	(F)CGGATCTCTTCCTGCTCGAC (R)GGTTCTTGGGCGTCTTGGAG
MMP14	(F)TCAACCCAGGACTACCTCCC (R)TCCTTCCTCTCGTAGGCAGT
SLIT2	(F)GTAAGTGCTTGGAGGGCCAT (R)TCCACTGCTGCATTCACAGT
SLIT3	(F)ACTGTCAACTTCGTGGGCAA (R)ATGCCGTTGTCCTTGTCAGT
TRPS1	(F)GGCAGCAGTCTGTCAAGGAA (R)GACGGCATTTGTAGCAGCTG
EFNB2	(F)TTCAGCCCTAACCTCTGGGG (R)AGTTCTGGACGTCTTGTTGGA
MET	(F)AGCTGCCAGTGAAGTGGATG (R)GGTCTGGGCAGTATTCGGG
WNT5A	(F)TCCGACATCGAAGGTGGAAC (R)CTCACCGCGTATGTGAAGGC
WNT5B	(F)CGGACAACGCATCTGTCTTTG (R)GGCTGATGGCGTTGACCA
WNT6	(F)CGAGAGTGCCAGTTCCAGTT (R)ATAGAACAGGCCTGCGTGAC
RSPO3	(F)AGTCCATGCACGAAGAAGGG (R)GTTGGGGGACACAGGTTACC
KREMEN	(F)CCCACCTCTGTCCTTCAACG (R)AGAGGACTTTGGAGGACCGG
CDC6	(F)TGCTCTTGATCAGGCAGTTGA (R) TCCAAGAGCCCTGAAAGTGAC
DNA2	(F)AGACAAATACCAAGGAAGGGACA (R)GCAACATTAAGACGTCGCCA
POLE2	(F) CGTCCGTTTTCCTAGCAGCA (R)CACTGGGCAGACATAAAGAGGT
TIPIN	(F) GCACCTGTTCCTGTACCTCC (R)TATGTGCCCAGTGCTCCATG
MAD2	(F) GCGCGTGCTTTTGTTTGTG (R)AGTAAAGGTTTCAGATGGATAT
BUB1	(F)AGAGCCCAGGAGACTTCACA (R)GTCGCCTGGGTACACTGTTT
BUBR1	(F)GGATGCCATTATCACAGGCT (R)TGCTGGTCCTCAGAGGTCTT
AUBORA-A	(F)GCTGGAGAGCTTAAAATTGCAG (R)TTTTGTAGGTCTCTTGGTATGTG
SOX2	(F)CAACCAGAAAAACAGCCCGG (R)TGTGCATCTTGGGGTTCTCC
S0X13	(F)AAGATCCTGCAAGCCTTCCC (R) CGCGCCTGTTCCTCATAGTA
TIF2	(F)TGTTTCCCTTAACGGCACCT (R)TGACACTCTTTTGGGATGCTGA
TGIF2	(F)TCTAGGTGAGGACGAAGGCC (R) TCTGAGGGGTAGGCGTTGTA
PRA2	(F) AAGCAGGGAACTTTGGTGGG (R) CTTCAGGTCTTGGACAAGCCT
HMGA1	(F)TCCAAGCAGGAAAAGGACGG (R)CCCCGAGGTCTCTTAGGTGT
PAX6	(F)TCACAAACACCTACAGCGCT (R) ATAACTCCGCCCATTCACCG

Table S3. The information of mRNA sequencing data quality control and genome alignment

Sample	Q30 reads (%)	Q30 bases (Mb)	Adapter (%)	GC content (%)	Clean reads (%)
Day 0	92.81%	5864102524	1.37 %	48.88 %	99%
Day 20	92.88%	5663194947	1.75 %	49.15 %	98.96%
Day 30	93.18%	6395957662	1.69 %	49.97 %	98.93%
